# Treatment of ovarian cancer ascites by intra-peritoneal injection of diphtheria toxin A chain-H19 vector: a case report

**DOI:** 10.1186/1752-1947-4-228

**Published:** 2010-07-27

**Authors:** Aya Mizrahi, Abraham Czerniak, Patricia Ohana, Smadar Amiur, Jennifer Gallula, Imad Matouk, Rasha Abu-lail, Tatiana Birman, Abraham Hochberg, Tally Levy

**Affiliations:** 1 The Hebrew University of Jerusalem, Biological Chemistry, Jerusalem, 91905, Israel; 2 Sheba Medical Center, Department of General and Hepatobiliary Surgery, Tel Hashomer, 52621, Israel; 3 E. Wolfson Medical Center, Genecology Oncology, Holon, 58100, Israel

## Abstract

**Introduction:**

Ovarian cancer ascitic fluid, which contains malignant cells, is usually present in women with an advanced stage disease. There are currently no effective therapies for the treatment of ovarian cancer ascitic fluid. We developed a new therapeutic strategy to target expression of the diphtheria toxin fragment A gene in ovarian tumor cells under the control of H19 regulatory sequences.

**Case presentation:**

A 64-year-old Caucasian woman was diagnosed with a stage IIIc epithelial ovarian cancer. She suffered from progressive disease, accumulation of malignant ascites that needed to be drained weekly, abdominal pain, vomiting, anorexia and severe weakness. Infusion of the diphtheria toxin A chain-H19 plasmid into the peritoneum of our patient resulted in complete resolution of the ascites with minimum adverse events.

**Conclusion:**

On the basis of this preliminary experience, we are currently conducting an extensive Phase I study on a larger number of patients in order to assess the safety and preliminary efficacy of this novel patient-oriented treatment approach.

## Introduction

H19 is a paternally imprinted, maternally expressed, oncofetal gene that has no protein product [1]. It is expressed at substantial levels in several different human tumor types, but is only marginally or not at all expressed in normal adult tissues [2,3]. Recent data suggested a role for H19 in promoting cancer progression, angiogenesis and metastasis [4].

Based on the results from our pre-clinical studies [5], we applied a tailored transcriptional regulatory sequence selection approach as a patient oriented DNA-based therapy. This approach to gene therapy of human cancer exploits the genetic and epigenetic alterations in cancer for targeting the expression of toxic genes. As a toxic gene, we chose the diphtheria toxin A chain (DT-A), which has suitable properties for achieving efficacious cancer cell killing [6]. The H19 promoter will drive the expression of the therapeutic protein, the DT-A chain. Diphtheria toxin is secreted from *Corynebacterium diphtheriae *as a single polypeptide chain containing two major domains: A chain (amino-terminal, 193 residues), which carries the active site for adenosine diphosphate (ADP)-ribosylation of elongation factor-2, and B chain (carboxyl-terminal, 342 residues), which promotes binding of toxin to cells and the entry of the A chain into the cytosolic compartment. The DT-A chain is a strong inhibitor of protein synthesis that catalyzes the ADP-ribosylation of diphthamide, a post-translationally modified histidine residue present in the elongation factor 2 (EF-2) where it catalyzes the transfer of ADP-ribose from nicotinamide adenine dinucleotide to EF-2, halting protein synthesis and killing the cell [7]. It should be noted that cellular uptake of the DT-A protein requires the B chain of diphtheria toxin, but is not present in the plasmid or in any normal cell [8-10]. Thus, in the absence of the DT-B chain, free DT-A cannot be taken up by neighboring cells. Unintended toxicity to other cells can be avoided by introducing the DT-A gene under the control of regulatory sequences of genes differentially expressed in tumours [11]. We constructed a plasmid in which the expression of DT-A is controlled by the transcriptional regulatory sequences of the H19 gene (DTA-H19).

Here we present evidence that treatment of a human patient with ovarian carcinoma using the DTA-H19 construct leads to a highly significant suppression of ascites progression with very limited apparent toxicity toward the host, indicating that these constructs have a high therapeutic potential and are very promising candidates for ovarian cancer therapy in humans.

## Case presentation

A 64-year-old Caucasian woman was diagnosed in 2002 with a stage IIIc epithelial ovarian cancer (EOC). She underwent optimal cytoreductive surgery that included bilateral salpingo-oophorectomy, hysterectomy, omentectomy, appendectomy, sigmoidectomy and partial stripping of the peritoneum from the right diaphragm. After six cycles of paclitaxel and carboplatin chemotherapy, she had no evidence of disease as evaluated by computed tomography (CT) scan or CA-125 levels. However, second-look laparoscopy revealed several small malignant nodules (less than 3 mm each) on the right diaphragm. Our patient received three courses of intra-peritoneal cisplatinum followed by four courses of docetaxel as maintenance treatment.

Two years later the patient experienced an intra-abdominal recurrence and was treated again with paclitaxel/carboplatin doublet. Due to stable disease after six courses of treatment she further received topotecan for eight courses followed by gemcitabine, doxil and etoposide. Despite this aggressive therapy, the woman suffered from progressive disease, accumulation of malignant ascites that needed to be drained weekly, abdominal pain, vomiting, anorexia and severe weakness.

**Figure 1:**
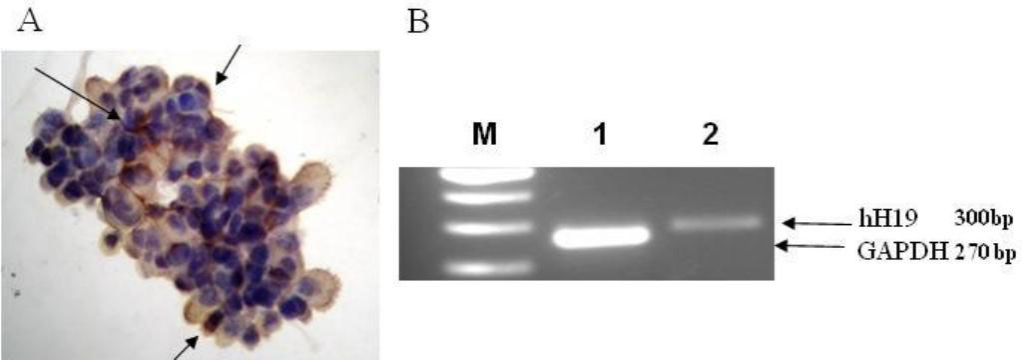
**The level of H19 transcript in RNA isolated from cells of ascites fluid of a woman patient, determined by RT-PCR or by ISH**. (A) 40× magnification of H19 transcripts in the isolated ascites cells determined by ISH analysis. The positive stained cells are marked by black arrows. (B) The H19 transcript in RNA extracted from ascites cells determined by RT-PCR analysis. M: 100 bp ladder, line 1 and 2 are the GADPH and H19 transcripts, respectively. The sizes of the GAPDH internal control and the human H19 PCR products (300 and 270 bp, respectively), are marked (black arrows).

Cells from our patient's ascites fluid were isolated for the detection of H19 RNA by *in situ *hybridization analyses (ISH) (Figure 1A) and by reverse transcriptase polymerase chain reaction (RT-PCR) analysis (Figure 1B). The isolated ascites cells were also positive for CA-125 expression as was tested by immunohistochemistry (IHC) analysis (data not shown). Figure 1 shows strong hybridization signals in the cytoplasm of our patient's ascites cells (Figure 1A). In addition, the RT-PCR analysis of RNA isolated from our patient's ascites cells also showed high levels of H19 RNA transcript (Figure 1B). Since almost all available treatment options had been administrated and high levels of H19 RNA transcripts were detected, she was offered a compassionate treatment with DTA-H19.

The study protocol was approved by the medical center's local ethics committees, and the ethical committee of the Israel Ministry of Health. The study was conducted according to the Helsinki Declaration and followed the principles of good clinical practice. Our patient provided written informed consent. Pre-treatment evaluation by positron emission tomography (PET)/CT showed an intra-peritoneal fluorodeoxyglucose (FDG) uptake without extra-peritoneal spread. An intra-peritoneal port-a-cath© (Smiths Medical) catheter was placed via laparoscopy during which extensive peritoneal spread of tumor was found especially in the upper abdomen.

Our patient was treated with weekly intra-peritoneal instillation of 80 mg of DTA-H19 vector diluted in 2000 cc NaCl 0.9%, and injected at a rate of 10 cc per minute (approximately 0.4 mg plasmid per minute). During the first hour after the treatment, our patient suffered from nausea, vomiting, fever and chills: blood, urine and ascetic fluid cultures were all negative for bacterial infection. Thus, in the next cycles she was pre-medicated with paracetamol, H_2 _blocker and anti-emetics and did not experience any further toxicities except for mild nausea.

At week four we increased the dose of the injected plasmid to 108 mg. Since there were no adverse events, we further increased the dose of the plasmid to 120 and 140 mg during weeks five to six and week seven, respectively (Table 1). Our patient was closely monitored for adverse events during the whole study period. Complete blood count, serum chemistry (kidney and liver functions) and urine analysis were performed. DT-A DNA was analyzed in the blood and in urine samples (at different times following the treatment) by quantitative real time PCR (QPCR) analysis.

**Table 1:** Ovarian cancer compassionate patient treatments summary.

Treatment number	Plasmid infused (mg)	Time between treatments	AEs*
1-3	80	One week	2 hours after 1st treatment: vomiting once, fever 38.3°c for 3 hours, chill, weakness. 2nd & 3rd treatments: Nausea, vomiting once

4	108	One week	Nausea

5-6	120	One week	Nausea

7	140	One week	Nausea

8-10	120	Two weeks	No AEs

*During or immediately after plasmid instillation, patient received medications to control the fever elevation, nausea and vomiting.

**Figure 2:**
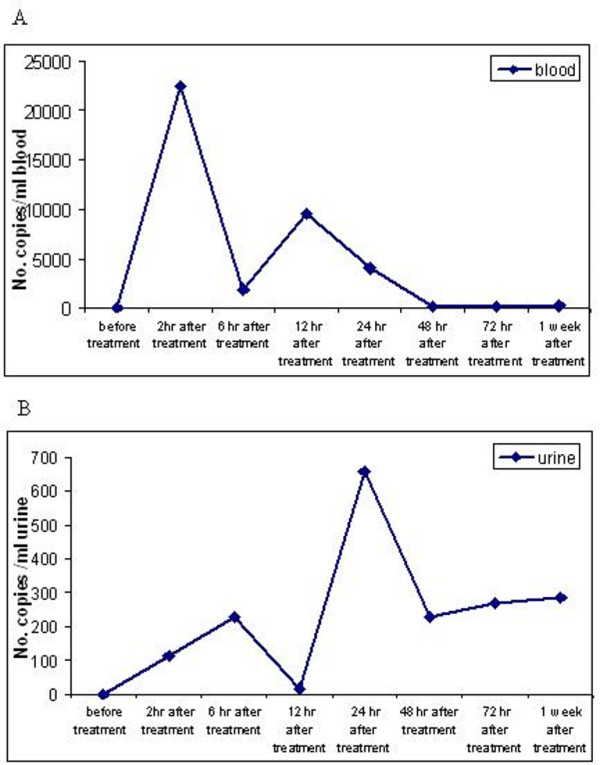
**Pharmacokinetics of DTA-H19 in blood and urine of the patient after the first treatment**. Blood and urine samples were collected before and after two, six, 12, 24, 48, 72 hours and one week from each of the treatments one to three. The urine samples were transferred into test tubes and frozen at -20°C until analysis. Blood samples were kept for 10 min at room temperature and then centrifuged for 10 min at 3500 rpm. The serum was transferred into a new test tube and kept at -20°C until analysis. Total DNA was isolated from blood and urine. The DNA samples were submitted to quantitative real time PCR analysis using specific probe. Panels A and B show the result for blood and urine samples, respectively.

Blood and urine were collected before and after the first three infusion cycles. As shown in Figure 2, two peaks of plasmid were detected after each treatment at two and 12 hours in the bloodstream and in the urine at six and 24 hours following plasmid infusion. The first peak was much higher than the second one. The plasmid DNA was still detectable by 48 hours after the infusion.

**Figure 3:**
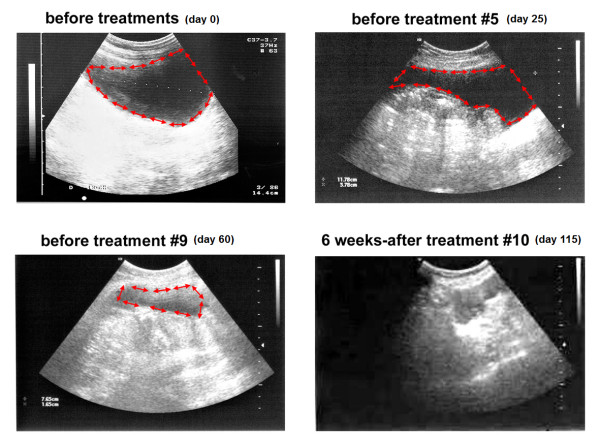
**Ultrasound image of peritoneal cavity from compassionate patient with ovarian carcinoma treated with intra-peritoneal injection of the DTA-H19 plasmid**. Ultrasound imaging was performed before each treatment to evaluate the amount of ascites fluid in our patient's peritoneum. A few representative figures of the imaging before the indicated treatments are presented. Red arrows indicate the limits around the area of ascites.

Ultrasound imaging performed before each treatment showed a reduction in the ascites volume. After ten weekly intra-peritoneal plasmid infusions our patient had complete resolution of her ascites (Figure 3).

Clinically, our patient experienced an excellent response with improvement in her general condition, appetite, weight gain and daily activities.

A sample of ascites fluid was collected before treatment number five and examined for malignant cells. Cells were isolated from ascites fluid using percoll gradient. Only a few tumor cells were observed microscopically.

CT and PET-CT scans were performed at six weeks and 3.5 months after the first treatment, respectively. Both scans showed that although there were still multiple intra-peritoneal areas of 2-Deoxy-2-[18F]fluoro-D-Glucose (FDG) uptake indicating the presence of cellular activity of the tumor, an arrest in disease progression and disappearance of ascites were noticed.

## Discussion

Following investigations of the efficiency, safety and toxicity of H19 DNA based therapy in animal models [5], we evaluated this novel intra-peritoneal treatment in a patient suffering from malignant ascites due to resistant EOC. A sample of ascites fluid containing malignant cells was collected and was submitted to histological and molecular diagnosis. Our patient was treated with the DTA-H19 plasmid administered intra-peritoneally after demonstrating high levels of H19 RNA in the ascites cells by ISH and RT-PCR analysis (Figure 1). The advantage of the intra-peritoneal treatment over the systemic treatment is to expose the cancer present within the peritoneal cavity to higher concentrations of a drug for more prolonged time periods than those obtained with systemic drug delivery. Taking into consideration the natural history of ovarian cancer, the anatomical and physiological characteristics of the peritoneal cavity and liver, direct intra-peritoneal drug delivery could result in a profound increase in the exposure of the cavity to certain anti-neoplastic drugs, while reducing systemic exposure and toxic effects [12].

The pharmacokinetics analysis of blood and urine collected before and after each treatment showed that plasmid DNA was detectable at two and 12 hours in the bloodstream and in the urine at six and 24 hours following plasmid infusion. The graph pattern shows two peaks of plasmid DNA in different time lines after infusion (Figure 2). This can be explained by the fact that some of the plasmid is transferred to the blood stream from the peritoneum which might be indicated by the first peak, and then it is transferred through the lymphatic system into the venous blood stream [13] which might be represented by the later second peak in the graph (Figure 2).

The main goal of the treatment was to reduce the amount of ascites fluid which compromised our patient's quality of life. We showed that the intra-peritoneal infusions were well tolerated and devoid of any detectable systemic toxicity related to the toxin vector administration. The amount of ascites fluid was reduced gradually during treatments and was completely abolished after treatment number five (Figure 3) as determined by clinical and ultrasound examination. It is important to note that no drainage of ascites fluid was performed until treatment number ten. We also tried to detect the plasmid in cells extracted from our patient's ascites, but three weeks after the first treatment, only few or no tumor cells could be isolated. The rapid reduction of cells in the ascites fluid might indicate that DTA was expressed from the DTA-H19 vector and caused cell death.

Imaging studies showed that although the ascites disappeared, our patient still had evidence of stable measurable disease. This can be explained by the fact that the penetration ability of the plasmid to deeper layers of the tumor is limited. This is in accordance with previous research in which large tumor masses essentially never regressed, even after ascites disappearance [14]. The main problem with regional anti-neoplastic therapy is probably the limited depth of penetration of drugs directly into the tumor (or normal tissues) by free-surface diffusion [13]. Keeping the safety of our patient was our first priority although, we are aware of the fact that a more efficient delivery approach should be developed.

## Conclusions

The novel DTA-H19 treatment achieved complete resolution of ascites and an excellent clinical response in a patient with end stage EOC resistant to chemotherapy. After seven months of plasmid therapy, the patient was still alive, had no ascites and resumed regular daily activities with an improved quality of life.

On the basis of this study we formed a platform for the design of an extensive Phase I study on a larger number of human patients to test the safety of this treatment.

The results obtained in the present study may represent the first step in a major breakthrough in the treatment of human OCAF. Data regarding the correlation between the level of H19 expression and the efficacy of the treatment should be collected during a Phase I and II clinical trials which are being planned. Based on the data collected during these future clinical trials we will be able to identify responders from non-responders in advance who are resistant to all known therapies, thereby avoiding treatment failure coupled with unnecessary suffering and cost.

## Abbreviations

CA-125: cancer antigen 125; DT-A: diphtheria toxin A chain; DTA-H19: vector expressing the DT-A gene under the control of H19 regulatory sequences; EOC: epithelial ovarian cancer; IHC: immunohistochemistry; ISH: *in situ *hybridization; OCAF: ovarian cancer ascites fluid; PCR: polymerase chain reaction; PEI: polyethylenimine; TCC: transitional cell carcinoma.

## Consent

Written informed consent was obtained from the patient's son for publication of this case report and accompanying images. A copy of the written consent is available for review by the Editor-in-Chief of this journal.

## Competing interests

The authors declare that they have no competing interests.

## Authors' contributions

AM participated in the design, coordination, and drafted the manuscript. AC participated in the study design and coordination. PO participated in design, coordination, data interpretation and drafted the manuscript. SA participated in study coordination. JG participated in raw data processing. IM participated in the *ex vivo *studies. RA participated in the PCR studies. VS participated in the *in vivo *studies. TB participated in the histology, IHC, ISH interpretation. AH conceived of the study, participated in design, interpretation of data, and critically revised the manuscript. TL participated in the study design and coordination, analyses of the ovarian ascites fluid.

All authors read and approved the final manuscript.
